# Laquinimod Modulates Human Astrocyte Function and Dampens Astrocyte-Induced Neurotoxicity during Inflammation

**DOI:** 10.3390/molecules25225403

**Published:** 2020-11-18

**Authors:** Emanuela Colombo, Rosaria Pascente, Daniela Triolo, Claudia Bassani, Anthea De Angelis, Francesca Ruffini, Linda Ottoboni, Giancarlo Comi, Gianvito Martino, Cinthia Farina

**Affiliations:** 1 Institute of Experimental Neurology (INSpe), Division of Neuroscience, IRCCS San Raffaele Hospital, 20132 Milan, Italy; colombo.emanuela@hsr.it (E.C.); rosaria.pascente@gmail.com (R.P.); triolo.daniela.estel@gmail.com (D.T.); bassani.claudia@hsr.it (C.B.); anthea.deangelis@gmail.com (A.D.A.); ruffini.francesca@hsr.it (F.R.); ottoboni.linda@hsr.it (L.O.); comi.giancarlo@hsr.it (G.C.); martino.gianvito@hsr.it (G.M.); 2 Faculty of Medicine and Surgery, Vita-Salute San Raffaele University, 20132 Milan, Italy

**Keywords:** AHR, astrocytes, glutamate transporters, laquinimod, neurodegeneration, NFκB

## Abstract

Astrocytes greatly participate to inflammatory and neurotoxic reactions occurring in neurodegenerative diseases and are valuable pharmacological targets to support neuroprotection. Here we used human astrocytes generated from reprogrammed fibroblasts as a cellular model to study the effect of the compound Laquinimod and its active metabolite de-Laquinimod on astrocyte functions and the astrocyte–neuron interaction. We show that human iAstrocytes expressed the receptor for the inflammatory mediator IL1 and responded to it via nuclear translocation of NFκB, an event that did not occur if cells were treated with Laquinimod, indicating a direct anti-inflammatory activity of the drug on the human astrocyte. Similarly, while exposure to IL1 downregulated glial glutamate transporters GLAST and GLT1, treatment with Laquinimod supported maintenance of physiological levels of these proteins despite the inflammatory milieu. Laquinimod also induced nuclear translocation of the aryl hydrocarbon receptor (AHR), suggesting that drug action was mediated by activation of the AHR pathway. However, the drug was effective despite AHR inhibition via CH223191, indicating that AHR signaling in the astrocyte is dispensable for drug responses. Finally, in vitro experiments with rat spinal neurons showed that laquinimod did not exert neuroprotection directly on the neuron but dampened astrocyte-induced neurodegeneration. Our findings indicate that fibroblast-derived human astrocytes represent a suitable model to study astrocyte–neuron crosstalk and demonstrate indirect, partial neuroprotective efficacy for laquinimod.

## 1. Introduction

Astrocytes are essential components of intrinsic innate immune responses of the central nervous system (CNS), and act as important mediators of neuronal damage in brain disorders [1,2]. They are able to sense any type of tissue insults and react activating a process defined as astrogliosis, characterized by a spectrum of molecular, cellular and functional changes that may either exacerbate tissue damage or contribute to CNS repair [3]. In fact, the fate of astrocytosis is governed by the balance between the activation of detrimental versus protective signaling pathways in astrocytes in response to multiple signals received from the inflamed environment [2]. Being an integral part of the blood brain barrier (BBB) and because expressing key immune receptors (Toll-Like receptors and cytokine receptors) [1,2,4], astrocytes respond to inflammatory factors, which spread through the BBB or are produced by activated microglia or infiltrating immune cells. Neuronal damage is then fostered by astrocyte release of neurotoxic mediators as reactive oxygen and nitrogen species [5,6,7], increased production of cytokines and chemokines [8] or excitotoxic levels of glutamate [9]. Thus, therapeutic manipulation of astrocytes is emerging as a promising neuroprotective option for neuroinflammatory diseases. Most information on astrocyte behavior has been obtained from in vivo and in vitro mouse models and requires validation with appropriate human models, as significant phenotypical and functional differences between human and rodent astrocytes may exist [10,11]. Human fibroblast-derived astrocytes (iAstrocytes) provide an unlimited cell source to explore the contributions of astrocytes to human diseases and test compounds modulating astrocyte activity and supporting neuroprotection. Here we exploited human iAstrocytes to investigate the effects of the anti-inflammatory quinoline-3-carboxamides derivatives (Q compounds) on glial activation and the glia–neuron interaction. Among Q compounds, laquinimod (Laq) is an experimental drug showing anti-inflammatory and neuroprotective properties in experimental neuroinflammation [12,13,14]. Our novel findings demonstrate that Laq and delaquinimod (de-Laq), the N-dealkylated active metabolite of Laq [15], dampen inflammatory signaling in astrocytes, preserve physiological astrocyte functions and prevent astrocyte-mediated neurodegeneration.

## 2. Results

### 2.1. Generation and Characterization of Human iAstrocytes

**Figure 1:**
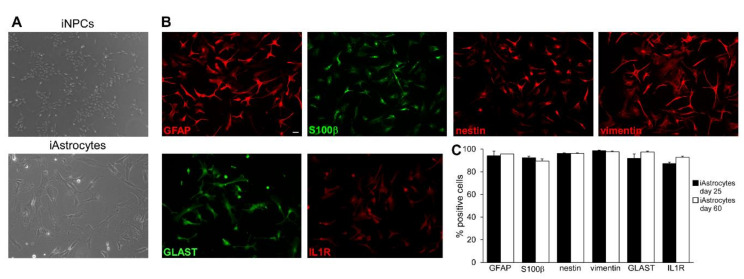
Generation and characterization of iPSC-derived astrocytes. (**A**) Phase contrast images showing cultured human iNPCs (upper panel) and human iAstrocytes (lower panel). (**B**) Representative immunofluorescence stainings for GFAP, S100β, nestin, vimentin, GLAST and IL1R in human iAstrocytes. (**C**) Percentage of iAstrocytes expressing astrocyte markers at two timepoints during differentiation. Graphs report data from two iAstrocyte cell lines. Bars represent SEM. Scale bar = 30 µm.

iAstrocytes were generated from induced pluripotent stem cells (iPSC)-derived neural precursor cells (iNPCs) as previously described [16]. iNPC differentiation induced cell size increase and typical morphology of astroglia (Figure 1A). As described [16,17], both iNPC and iAstrocytes expressed typical markers as GFAP, S100ß, nestin, vimentin and GLAST as detected by immunofluorescence experiments (Figure 1B,C and Figure S1). Astrocytes displayed strong expression of Interleukin 1 receptor (IL1R; Figure 1B,C), an inflammatory receptor upregulated under neuroinflammation [4], indicating that our in vitro human cell model could be responsive to inflammatory mediators.

### 2.2. Laq Blocks the Inflammatory Activation of iAstrocytes and Supports Maintenance of Glutamate Transporters

**Figure 2:**
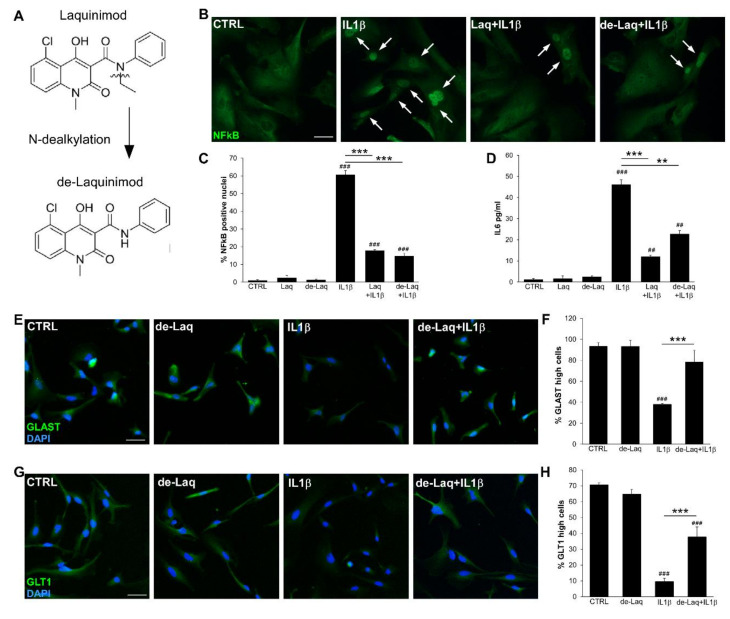
Laq inhibits NFκB nuclear translocation and maintains glutamate transporters expression in astrocytes exposed to IL1β. (**A**) Chemical structures of laquinimod and de-laquinimod. (**B**) Representative immunofluorescence stainings for NFκB in human iAstrocytes exposed to drugs and/or IL1β. White arrows highlight positive nuclei. (**C**) The graph reports the percentage of NFκB positive nuclei under distinct conditions. (**D**) IL6 protein levels in supernatants from CTRL or IL1β-stimulated iAstrocytes after pre-exposure to drugs detected by the ELISA assay. (**E**–**H**) Representative immunofluorescence stainings for GLAST (**E**) and GLT1 (**G**) in human iAstrocytes and relative quantifications (**F**,**H**) under distinct conditions. For drug treatment, cells were exposed to 250 nM laquinimod or 100 nM de-laquinimod. DAPI was used for nuclear staining. Data are shown as mean ± SD of a representative experiment out of 2–3 independent experiments. Scale bars: 30 µm. # indicates statistical significance versus CTRL. * above the bar indicates statistical significance between specific experimental conditions. ** *p* < 0.01, *** *p* < 0.001, ^##^ *p* < 0.01, ^###^ *p* < 0.001.

Nuclear factor kappa B (NFκB) is a key transcription factor in cytokine signaling, and astroglial NFκB drives the amplification of inflammatory and neurodegenerative processes [2,18,19]. To address the effect of Laq and de-Laq (Figure 2A) on astrocyte activation, we verified the impact of drug treatment on nuclear translocation of NFκB. As shown in Figure 2B, several nuclei were positive for NFκB when cells were stimulated with the inflammatory cytokine IL1β (CTRL vs. IL1β *p* < 0.001). On the contrary, when astrocytes were previously exposed for 4 h to Laq or de-Laq, IL1β-induced NFκB nuclear translocation was blocked (Figure 2B,C; IL1β vs. Laq + IL1β or de-Laq + IL1β *p* < 0.001).

NFκB activation in astrocytes induces the expression of various proinflammatory cytokines, including IL6 [20]. As astrocytic IL-6 has a key role in neuroinflammation [21,22] we evaluated drug effects on the production of this cytokine by resting and IL1β-activated astrocytes. IL1β triggered IL6 release (Figure 2D; CTRL vs. IL1β *p* < 0.001), but exposure to Laq or de-Laq significantly mitigated these levels (Figure 2D; IL1β vs. Laq+ IL1β or de-Laq + IL1β *p* < 0.001 and *p* = 0.0052 respectively).

Extracellular glutamate buffering below neurotoxic levels is a critical physiological function of astrocytes performed via two glutamate transporters, GLT1 and GLAST [23]. To address glutamate transporter expression in glia cells, iAstrocytes were incubated with de-Laq or vehicle, then eventually exposed for 24 h to IL1β, and checked for glutamate transporter levels by immunofluorescence. GLAST and GLT1 were expressed at a high level on the majority of resting iAstrocytes but resulted strongly downregulated in cells exposed to IL1β (Figure 2E–H; CTRL vs. IL1β *p* < 0.001). Notably, when cells were pretreated with de-Laq, the fraction of GLAST (Figure 2E,F) and GLT1 (Figure 2G,H) highly expressing astrocytes remained high (IL1β vs. de-Laq+ IL1β *p* < 0.001). Similar to its active metabolite, exposure to Laq blocked IL1β-induced GLAST downregulation when used at 250 nM but not at 100 nM (Figure S2; IL1β vs. 250 nM Laq+ IL1β *p* = 0.0113). All together, these data suggest that Q compounds may hamper the activation of inflammatory signaling cascades in glia cells and maintain physiological astrocyte functions.

### 2.3. Laq Effects on Human iAstrocytes Are Independent from AHR Signaling

**Figure 3:**
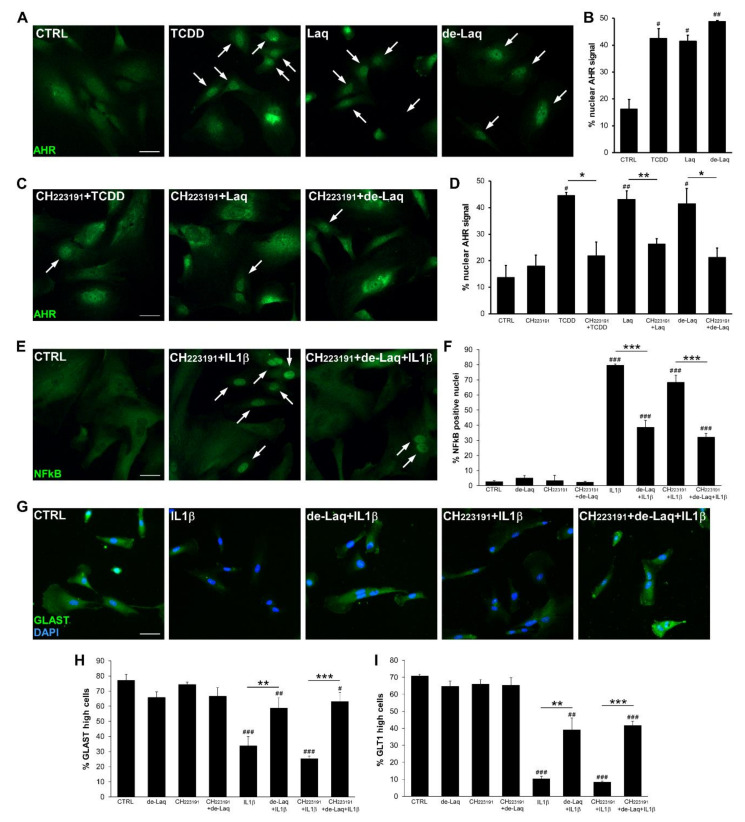
Laq activates nuclear AHR translocation but its effects on astrocyte functions are independent from AHR activity. (**A**,**C**) Representative immunofluorescence images for AHR in human iAstrocytes under distinct conditions and relative quantifications (**B**,**D**). (**E**,**F**) Immunofluorescence stainings for NFκB upon CH223191 treatment (**E**) and relative quantifications (**F**). (**G**) Images depicting GLAST stainings in human iAstrocytes and (**H**,**I**) frequency of GLAST (**H**) or GLT1 (**I**) highly expressing cells under the different experimental conditions. For drug treatment, cells were exposed to 250 nM laquinimod, 100 nM de-laquinimod and/or 10 µM CH223191. DAPI was used for nuclear staining. Data are shown as mean ± SD of a representative experiment out of 2–3 independent experiments. Scale bars: 30 µm. # indicates statistical significance versus CTRL. * above the bar indicates statistical significance between specific experimental conditions. * *p* < 0.05, ** *p* < 0.01, *** *p* < 0.001, ^#^ *p* < 0.05, ^##^ *p* < 0.01, ^###^ *p* < 0.001.

In vivo studies indicate that Laq action may be mediated by activation of the aryl hydrocarbon receptor (AHR) signaling pathway [24]. AHR is a ligand-activated transcription factor critical for responses to environmental stimuli including toxic chemicals like 2,3,7,8-tetrachlorodibenzo-p-dioxin (TCDD) [25]. To verify whether Laq activated directly AHR signaling in astrocytes, we assessed nuclear translocation of AHR in response to the drugs, or TCDD as a positive control, by immunofluorescence. Under resting conditions, few iAstrocytes displayed nuclear AHR expression (Figure 3A), however exposure to Laq or de-Laq significantly increased AHR nuclear signal (Figure 3A,B; CTRL vs. Laq *p* = 0.0032; CTRL vs. de-Laq *p* = 0.0108). CH223191, a dioxin ligand-selective antagonist of AHR [26], inhibited AHR nuclear translocation induced by TCDD and, most importantly, by Laq and de-Laq, demonstrating the specific effect of the drugs on AHR function (Figure 3C,D; Laq vs. CH223191 + Laq *p* = 0.0077; de-Laq vs. CH223191 + de-Laq *p* = 0.0226). To verify whether drug action on astrocyte function depended on AHR signaling, we repeated NFκB and glutamate transporter assays in human iAstrocytes after exposure to CH223191. As shown in Figure 3E,F, de-Laq blocked IL1β-induced NFkB translocation even in AHR-defective cells (CH223191 + IL1β vs. CH223191 + de-Laq + IL1β *p* < 0.001). Similarly, AHR inhibition via CH223191 did not dampen de-Laq capability to prevent GLAST and GLT1 downregulation in IL1β-treated astrocytes (Figure 3G–I; CH223191 + IL1β vs. CH223191 + de-Laq+ IL1β *p* < 0.001). Overall, these experiments indicate that Laq compounds modulate glial function independently from the activation of AHR.

### 2.4. Laq Hampers Astrocyte-Dependent Neurotoxicity but Not Direct Inflammation-Induced Neurodegeneration

**Figure 4:**
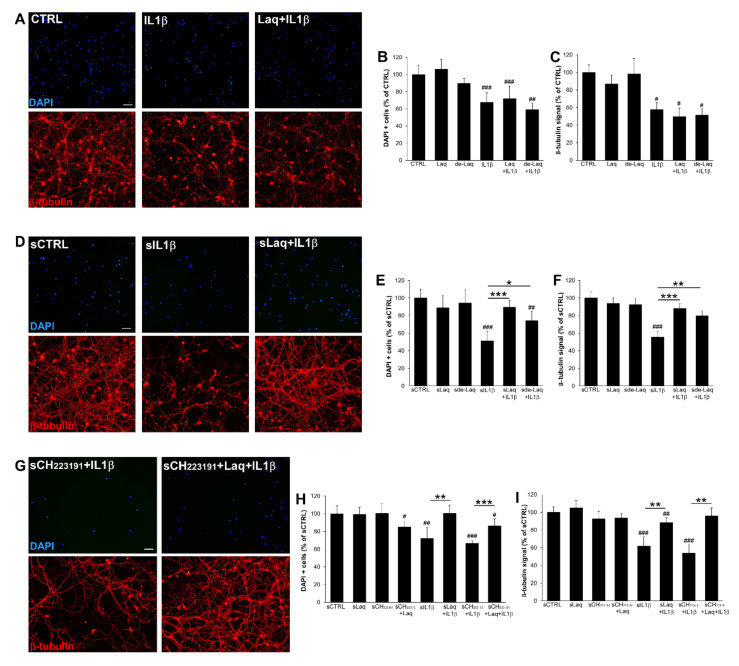
Laq blocks neurodegeneration induced by astrocyte responses to IL1β. (**A**) Representative immunofluorescence stainings for DAPI (upper panels) and β-tubulin (lower panels) in neuronal cultures exposed to IL1β alone or pre-treated with Laq. (**B**,**C**) Quantification of cell number (**B**) and β-tubulin signal (**C**) expressed as the percentage with respect to control cultures. (**D**) Representative images showing DAPI (upper panels) and β-tubulin (lower panels) stainings in neuronal cultures exposure to distinct iAstrocyte-conditioned media and relative quantification (**E**,**F**). (**G**–**I**) Representative immunofluorescence images for neuronal cultures exposed to conditioned media from iAstrocytes stimulated in the presence of the AHR antagonist CH22319. For drug treatment, cells were exposed to 250 nM laquinimod, 100 nM de-laquinimod and/or 10 µM CH223191. Graphs show cumulative results from 2 independent experiments. Data are represented as mean ± SEM. Scale bars: 50 µm. # indicates statistical significance versus CTRL. * above the bar indicates statistical significance between specific experimental conditions. * *p* < 0.05, ** *p* < 0.01, *** *p* < 0.001, ^#^ *p* < 0.05, ^##^ *p* < 0.01, ^###^ *p* < 0.001.

To address whether the drugs protect neurons from inflammation-induced neurodegeneration, rat spinal neurons were exposed to IL1β in the presence or absence of the compounds and assessed for cell number and morphology via DAPI and β-tubulin stainings. As previously shown [5], IL1β strongly reduced the number of neurons in culture and affected neuronal network integrity (Figure 4A; CTRL vs. IL1β: % DAPI positive cells *p* < 0.001, β-tubulin signal *p* = 0.04). Similarly, exposure of neurons to the drugs did not protect cells from IL1β-induced fragmentation of the neuronal network and death (Figure 4A–C). To study the effects of Laq and de-Laq on astrocyte–neuron interactions, in a second set of experiments we exposed human iAstrocytes to the compounds and then to IL1β for 8 h, changed the medium to remove stimuli, and collected the supernatants after a further 24 h culture. Astrocyte-conditioned media were then offered to spinal neurons. While supernatants from control cultures (sCTRL) did not affect network integrity and neuronal survival, conditioned media from human iAstrocytes exposed to IL1β triggered robust degenerative responses (Figure 4D–F; sCTRL vs. sIL1β: % DAPI positive cells and β-tubulin signal *p* < 0.001). However, when astrocyte media were generated in the presence of Laq (Figure 4E; sIL1β vs. sLaq + IL1β: % DAPI positive cells and β-tubulin signal *p* < 0.001) or de-Laq (Figure 4F; sIL1β vs. sde-Laq + IL1β: % DAPI positive cells *p* = 0.0123 and β-tubulin signal *p* = 0.0012), their addition to spinal neurons did not trigger neurodegeneration despite astrocyte exposure to IL1β. Moreover, blockade of AHR signaling in astrocytes via CH223191 did not reduce the neuroprotective action mediated by the drugs in glia cells as astrocyte media from Laq-treated cells did not result in being toxic to neurons (Figure 4G–I), thus confirming that AHR activation is dispensable for the modulation of the astrocyte response to inflammation by Laq.

## 3. Discussion

In this study we used an in vitro human cell model constituted by fibroblast-derived astrocytes to investigate the action of Laq, or its active metabolite de-Laq, on astrocyte activity during inflammation. We demonstrated that Laq directly hampered inflammation-induced NFκB activation in astrocytes and restored physiological glial functions due to the maintenance of the expression of glutamate transporters, and that AHR signaling was dispensable for drug action. Moreover, we showed that Laq protected from astrocyte-induced neurodegeneration but not from direct inflammatory neuronal damage.

Laq is an anti-inflammatory drug with proven efficacy in various neuroinflammatory models. Laq treatment reduces CNS demyelination, acute axonal damage, microglia activation and astrogliosis in mice with experimental autoimmune encephalomyelitis (EAE) [12,14,27] or under diet with the demyelinating agent cuprizone [13,28], two demyelinating models for multiple sclerosis (MS). Furthermore, Laq administration improves motor functions and reverses alterations in myelin sheath thickness in the experimental models for Huntington disease (HD) [29,30]. In vitro studies indicate that Laq regulates distinct immune cell functions, as monocyte migration [31], anti-inflammatory myeloid and lymphocytic cell differentiation [32] and antigen presentation [33]. Laq can cross the blood–brain barrier [34], thus it may potentially exert functions on CNS resident cells. Experiment on human cell cultures indicate that Laq decreases cytokine release from LPS-stimulated human microglia [35], and inhibits NFκB activation and the expression of proinflammatory cytokines in IL1-stimulated human astrocytes [13,28]. Our in vitro data obtained with iAstrocytes corroborate the evidence that Laq treatment inhibits glial NFκB activation under inflammatory conditions and extend it to de-Laq.

Glutamate is the main excitatory neurotransmitter in the CNS, and is cleared from the extracellular space by astrocytes via the glutamate transporters GLAST and GLT1 [36]. Neuroinflammation however, lowers glutamate transporters in vivo and in vitro [16,37,38,39], thus generating high glutamate concentrations in the extracellular cleft, which are toxic to neurons [40]. Laq can restore basal striatal glutamatergic currents if preventively administered to EAE mice [14] and ameliorate cerebellar glutamatergic transmission ex vivo via GLT1 expression in the tissue [41], however the mechanisms leading to these results have not been explored. Here we demonstrated the direct action of Laq on the maintenance of glutamate transporters in human astrocytes under inflammatory conditions, thus implying that this class of compounds may support physiological astrocyte functions.

Laq is a known activator of AHR [24,42]. Upon ligand binding, AHR translocates to the nucleus and regulates the expression of diverse target genes, including those involved in the activation of anti-inflammatory reactions, suppression of proinflammatory cytokines, production of reactive oxygen species and immune cell differentiation [43,44]. Regarding the role of AHR signaling within the CNS, AHR drives protective astrocyte responses to inflammation, as it acts as a negative regulator of NFκB activation and animals lacking astrocyte AHR develop more severe EAE [45]. Laq ameliorates disease severity in EAE mice and this effect is abolished in AHR knockout animals [24,42]. Nevertheless, Laq treatment may preserve axonal integrity and myelin both in control and AHR knockout EAE animals, demonstrating the existence of alternative pathways to AHR signaling mediating protective drug responses in the CNS [42]. Accordingly, treatment with Laq of bone marrow chimera EAE mice demonstrated a major role for AHR signaling in peripheral immune compartment and not CNS compartment in mediating Laq efficacy [24]. Our experiments confirm AHR as a target of Q compounds in human glia cells, but clearly demonstrate that the activation of this transcription factor is not a crucial event in the regulation of astrocyte function by the drug, as the AHR signaling blockade with a specific inhibitor does not compromise Laq efficacy in all our in vitro assays. We know that under the same experimental conditions astrocytes respond to IL1 with the release of nitric oxide, which may induce neurodegeneration [5], and that other types of drugs may lead to neuroprotection via blockade of nitric oxide production by activated astrocytes [4]. Nitric oxide synthesis depends on NFKB activation [46], thus we speculated that NFKB inhibition by Laq might also limit NO release.

Treatment with Laq has been tested in clinical trials for MS and HD but failed to meet the primary endpoints [47,48,49,50]. Anyway, these trials collected evidence for neuroprotection in treated subjects, as a reduction of brain atrophy and astrocytosis were observed [51,52,53,54]. Our in vitro experiments demonstrate that Laq cannot support direct rescue of neurons exposed to the inflammatory mediator IL1β, however it can modulate astrocyte function so that glia cells do not release factors triggering neurodegeneration. These evidence may explain the limited efficacy of the drug in human and pave the way to future studies combining drugs with distinct, complementary mechanisms of action for the treatment of neurodegenerative disorders.

In conclusion, our findings indicate fibroblast-derived human astrocytes as a suitable model to study drug action on astrocyte–neuron crosstalk. Moreover, we demonstrated an effect for laquinimod on astrocyte function, which results in indirect neuroprotection.

## 4. Materials and Methods

### 4.1. Fibroblast Reprogramming and Differentiation into iAstrocytes

Human fibroblast-derived astrocytes were produced as described [16]. Human skin biopsies were obtained from two healthy subjects after signing of informed consent approved by the Ethics Committee of Ospedale San Raffaele (Milan, Italy) (Prot. n. Banca-INSPE). All experiments were carried out following the rules of the Declaration of Helsinki of 1975 and performed in accordance with relevant guidelines and regulations. Human fibroblasts were reprogrammed into induced pluripotent stem (iPS) cells with the Sendai virus technology (CytoTune-iPS Sendai Reprogramming Kit, Thermo Fisher Scientific, Waltham, MA, USA). iPS cells were differentiated into neural precursor cells (iNPCs) via dual SMAD inhibition (SB431542/Dorsomorphin)/Hedgehog pathway activation (SAG/Purmorphamine)/WNT pathway activator (CHIR99021). iNPCs were maintained in a proliferation medium as described in [55] or differentiated into astrocytes with DMEM supplemented with 1% antibiotics, 200 mM L-glutamine, 100 mM sodium pyruvate (Thermo Fisher Scientific, Waltham, MA, USA), 10% FCS and 0.3% N2. Astrocytes were allowed to differentiate for several weeks, and checked for morphology and marker expression at different time points. Phase contrast images for morphologic assessment were obtained at Leica DMIL LED microscope.

### 4.2. Human iAstrocyte Assays

To detect NFkB activation and glutamate transporter expression, human iAstrocytes were eventually incubated with 10 µM CH223191 (Sigma, Milan, Italy) or vehicle (PBS or DMSO max 0.2% v/v) for 1 h, then exposed to 100 or 250 nM laquinimod (TEVA Pharmaceutical Industries, Rho, Milan, Italy), 100 nM de-laquinimod (TEVA Pharmaceutical Industries) or a vehicle for an additional 4 h, and finally stimulated with 10 ng/mL IL1β (Thermo Fisher Scientific) for 30 min for the NFκB assay or 24 h for the glutamate transporter assay. For the AHR assay, cells were exposed for 1 h to CH223191 or the vehicle and then stimulated with laquinimod or de-laquinimod or 1 nM 2,3,7,8-tetrachlorodibenzo-p-dioxin (LGC Standards, Sesto San Giovanni, Milan, Italy) for 60 min. Cells were finally processed for immunofluorescence and stained with appropriate primary antibodies.

For the generation of astrocyte-conditioned supernatants for IL6 detection, iAstrocytes were preincubated in serum-free media for 4 h with drugs, and then exposed to IL1β for 24 h. Supernatants were then collected and centrifuged.

For the generation of astrocyte-conditioned media iAstrocytes were eventually preincubated with 10 µM CH223191 or a vehicle for 1 h, stimulated for 4 h with drugs, and then exposed to IL1β for 24 h. Medium was then replaced with fresh neuronal medium and, after an additional 24 h of culture, supernatants were collected, centrifuged to remove cell debris and stored at −80 °C. Before addition to primary neurons, astrocyte supernatants were diluted 1:4 with medium.

### 4.3. Generation and Treatment of Primary Spinal Neurons

All procedures involving animals were conducted in accordance with relevant guidelines and regulations. All experimental protocols were approved by the local Animal Ethical Committee and the Italian General Direction for Animal Health at the Ministry of Health (Rome, Italy) (Approval Number: 950). Primary spinal neurons were obtained from 16-day-old Sprague Dawley rat embryos as described [4,5,16]. Briefly, embryonal spinal cords, depleted of spinal root ganglia, were dissected, carefully minced and digested for 15 min at 37 °C with 500 µg/mL DNAsi I (Roche, Monza Brianza, Italy) and 0.25% trypsin (Thermo Fisher Scientific, Monza Brianza, Italy) in L-15 medium (Thermo Fisher Scientific, Monza Brianza, Italy) supplemented with antibiotics. After digestion, tissue homogenate was washed 3 times with L-15 medium and finally cultured in Neurobasal medium (Thermo Fisher Scientific, Monza Brianza, Italy) supplemented with 10 ng/mL glial cell-derived neurotrophic factor (GDNF; Sigma), 20 ng/mL fibroblast growth factor (FGF; Peprotech, London, UK), 50 µg/mL insulin (Sigma), B27 supplement (Thermo Fisher Scientific), 1% FCS (Euroclone, Pero, Milan, Italy) and 10 mM Glucose. Cells were seeded on poly-D-lysine and collagen (both from Sigma) coated glass coverslips. After 24 h, 15 µM cytosine b-D-arabinofurnoside (AraC; Sigma) was added to cultures and left for 4 days to eliminate contaminating microglia cells, astrocytes and oligodendrocytes. Neurons were exposed for 4 h to laquinimod or de-laquinimod, then stimulated with vehicle or 10 ng/mL IL1β for 24 h. Alternatively, spinal neurons were stimulated with astrocyte conditioned media for 8 h prepared as described above, then processed for immunofluorescence and stained with a monoclonal antibody against β-tubulin. For assessment of neuronal counts, the numbers of DAPI positive nuclei were quantified and reported as a percentage of control. Neuronal network was measured by β-tubulin signal and expressed as a percentage of control.

### 4.4. Immunofluorescence Experiments

Immunofluorescence experiments were performed as previously described [16]. Astrocytes or neurons were plated on coverslips, fixed with 4% PFA or MetOH, permeabilized with 0.2% Triton X-100 (Merck Millipore, Milan, Italy), blocked in PBS+ 1% BSA (Merck Millipore, Milan, Italy) + 5% FCS and stained with primary antibodies. Then, cells were incubated with appropriate species-specific Alexa Fluor 488/594-conjugated secondary antibodies (Thermo Fisher Scientific, Monza Brianza, Italy), counterstained with 4’,6-diamidino-2-phenylindole (DAPI, Sigma) and mounted with fluorescent mounting medium (Agilent, Cernusco sul Naviglio, Milan, Italy). The following primary antibodies were used: rabbit anti-GFAP (Agilent), mouse antinestin (Merck Millipore, Milan, Italy), mouse antivimentin (Abcam, Cambridge, UK), rabbit anti-S100β (Abcam), mouse anti-IL1R (R&D, Minneapolis, MN, USA), rabbit anti-NFκB p65 (Abcam), rabbit anti-GLAST (Abcam), guinea pig anti-GLT1 (Merck Millipore), rabbit anti-AHR (US Biological, Salem, MA, USA) and mouse anti Neuronal Class III β-Tubulin (Covance, Milan, Italy). The following secondary antibodies were used: Alexa Fluor 488 donkey antirabbit IgG (H + L), Alexa Fluor 594 donkey antirabbit IgG (H + L), Alexa Fluor 488 donkey antimouse IgG (H + L), Alexa Fluor 594 donkey antimouse IgG (H + L) and Alexa Fluor 488 goat antiguinea pig IgG (H + L; all from Thermo Fisher Scientific). Fluorescence images were captured at fluorescence microscope (DM5500B, Leica, Buccinasco, Milan, Italy). LASX and ImageJ (download at: http://rsbweb.nih.gov/ij/) software were used for image acquisition and analysis respectively. To quantify nuclear NFκB and AHR, DAPI images were converted to 8-bit, and regions of interest (ROIs) were generated to select (DAPI positive) nuclei (an example of the analysis strategy for AHR is depicted in Figure S3). Then ROIs were applied to the corresponding NFκB or AHR images and fluorescence thresholds were fixed on the unstimulated condition. For AHR assay the area of the AHR nuclear signal was quantified and expressed as the percentage of total nuclear area. For the NFκB assay the number of positive nuclei was expressed as a percentage of total cells. To quantify cellular GLAST and GLT1, a threshold was set to identify the fraction of highly fluorescent astrocytes under the distinct conditions.

### 4.5. ELISA Assay

IL6 levels were measured in iAstrocyte supernatants using the Rat antihuman IL-6 and biotin rat antihuman IL-6 (BD Biosciences, Buccinasco, Milan, Italy) as capture and detection antibodies, respectively. The avidin-conjugated horseradish peroxidase (HRP) and 3,3’,5,5’-tetramethylbenzidine (TMB) microwell peroxidase substrate system (both from Thermo Fisher Scientific, Monza Brianza, Italy) were used for quantification. Colorimetric read-out was analyzed at the Epoch microvolume spectrophotometer system (Biotek, Bernareggio, Italy).

### 4.6. Statistical Analyses

Data in figures are presented as the mean ± standard deviation (SD) or standard error of the mean (SEM) as indicated in figure legends. The exact number of independent experiments performed is reported in figure legends. A one way ANOVA test was performed to compare means. All *p*-values were two-sided and subjected to a significance level of 0.05. In figures, symbols (* and #) denote statistical significance as * *p* < 0.05; ** *p* < 0.01; *** *p* < 0.001. Statistical analyses were performed in Excel or GraphPad Prism.

## Supplementary Materials

The following are available online. Figure S1. Characterization of iPSC-NPCs; Figure S2: Dose-dependent efficacy of Laquinimod in the maintenance of GLAST expression; Figure S3. Analysis strategy for quantification of AHR nuclear signal.
