# Supplementary material for: Laquinimod Modulates Human Astrocyte Function and Dampens Astrocyte-Induced Neurotoxicity during Inflammation
Source: Molecules. 2020 Nov 18;25(22):5403. doi: 10.3390/molecules25225403 (PMC7699283; doi:10.3390/molecules25225403)
Supplement: Supplementary file 1 [file molecules-25-05403-s001.pdf]

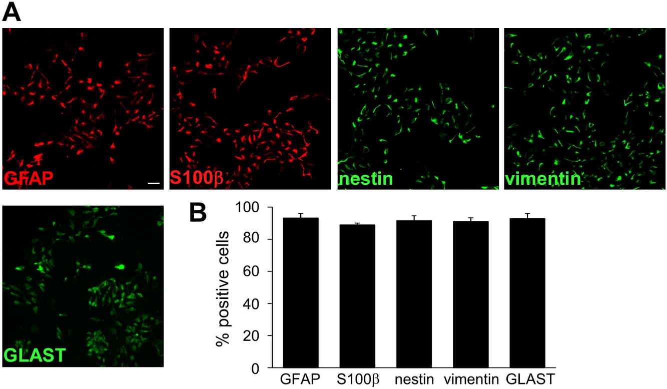


**Figure S1. Characterization of iPSC-NPCs.** (A) Representative immunofluorescence stainings for GFAP, S100ß, nestin, vimentin, and GLAST in human iNPCs and relative quantifications (B). Graphs report data from two iNPC cell lines. Bars represent SEM. Scale bar = 30μm.

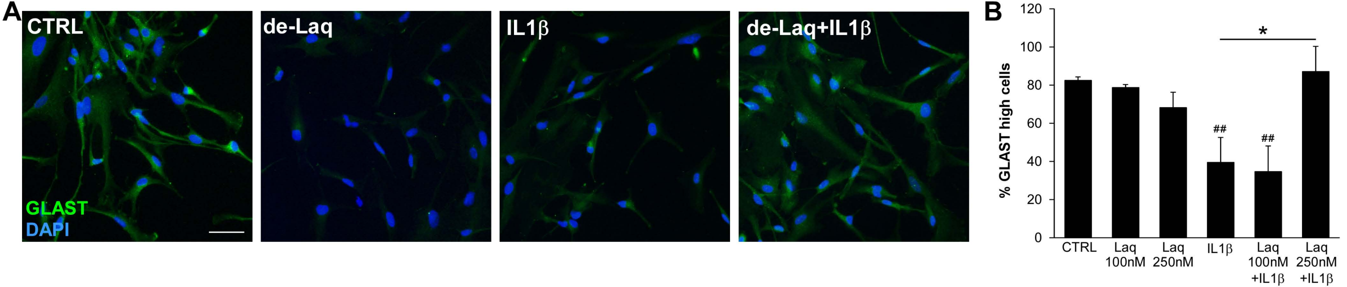


**Figure S2: Dose-dependent efficacy of Laquinimod in the maintenance of GLAST expression.** (A) Representative immunofluorescence stainings for GLAST In human iAstrocytes exposed to 100 nM or 250 nM Laquinimod and (B) relative quantifications. DAPI was used for nuclear staining. Data are shown as mean ± SD of a representative experiment out of 2independent experiments. Scale bars: 30 µm. # indicate statistical significance versus CTRL. * above bar indicate statistical significance between treatments. *= p < 0.05, **= p< 0.01.

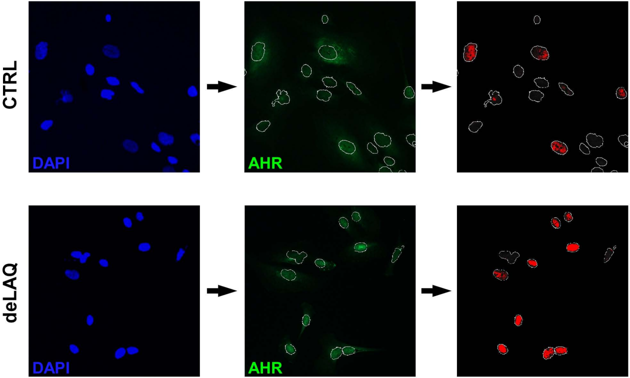


**Figure S3. Analysis strategy for quantification of AHR nuclear signal.**

Analysis strategy for quantification of AHR nuclear signal. Regions of interest (ROIs) were generated on DAPI images (left panels) to select nuclei. The same ROIs were then applied to the corresponding AHR image (middle panels) and area of AHR nuclear signal was quantified and expressed as percentage of total nuclear area.
